# A Simple, Low-Cost Conductive Composite Material for 3D Printing of Electronic Sensors

**DOI:** 10.1371/journal.pone.0049365

**Published:** 2012-11-21

**Authors:** Simon J. Leigh, Robert J. Bradley, Christopher P. Purssell, Duncan R. Billson, David A. Hutchins

**Affiliations:** 1 School of Engineering, University of Warwick, Coventry, Warwickshire, United Kingdom; 2 GKN Aerospace, Filton, Bristol, United Kingdom; Florida International University, United States of America

## Abstract

3D printing technology can produce complex objects directly from computer aided digital designs. The technology has traditionally been used by large companies to produce fit and form concept prototypes (‘*rapid prototyping*’) before production. In recent years however there has been a move to adopt the technology as full-scale manufacturing solution. The advent of low-cost, desktop 3D printers such as the RepRap and Fab@Home has meant a wider user base are now able to have access to desktop manufacturing platforms enabling them to produce highly customised products for personal use and sale. This uptake in usage has been coupled with a demand for printing technology and materials able to print functional elements such as electronic sensors. Here we present formulation of a simple conductive thermoplastic composite we term ‘carbomorph’ and demonstrate how it can be used in an unmodified low-cost 3D printer to print electronic sensors able to sense mechanical flexing and capacitance changes. We show how this capability can be used to produce custom sensing devices and user interface devices along with printed objects with embedded sensing capability. This advance in low-cost 3D printing with offer a new paradigm in the 3D printing field with printed sensors and electronics embedded inside 3D printed objects in a single build process without requiring complex or expensive materials incorporating additives such as carbon nanotubes.

## Introduction

3D printing (3DP) is a term to describe technology used for the rapid production of 3D objects directly from digital computer aided design (CAD) files [1] The 3D printing process allows 3D objects to be fabricated in a bottom-up, additive fashion directly from digital designs, with no milling or molding. It can be likened to clicking on the print button on a computer and sending a digital file, such as a letter, to a printer sitting on an office desk. The difference is that in a 3D printer the material or ink is deposited in successive, thin layers on top of each other to build-up a solid 3D object. The layers are defined by software that takes a series of digital cross-sections through a computer-aided design. Descriptions of the slices are then sent to the 3D printer to construct the respective layers. The layers can be constructed in a number of ways depending on the 3D printer being used. Powder can be spread onto a tray and then solidified in the required pattern with an amount of a liquid binder [2] or by sintering with a laser [3] or an electron beam [4]. Some machines carry out 3D lithographic processes using light and photosensitive resins [5] and others deposit filaments of molten plastic [6]. However each layer is constructed, after the layer is complete the build surface is moved by a fraction of a millimetre and the next layer of material is added. The most prolific technology used in low-cost 3D printers such as the RepRap [7] is Fused Deposition Modeling (FDM) or Fused Filament Modeling (FFM). FFM machines work on the simple principle of extruding a thin (sub 1 mm) filament of molten thermoplastic (normally from a feedstock of larger filament or powder) through a heated nozzle onto a room temperature or heated build platform. The printed filament network cools and adheres to the previously deposited layers to build up a solid 3D object.

Product designers have used 3D printing for over a decade to make limited-functionality models and prototypes before embarking upon the expensive business of fabricating tooling to produce a final product. More recently however, the technology has found greater appeal in more final-product based manufacturing across diverse fields from medical implants [8] right through to the artistic and creative industries [9]. With the proliferation of 3D printers such as the Reprap and Fab@Home [10] 3DP has also facilitated an individualised or personalised approach to manufacturing, where objects can be customised and produced by an individual to their own specifications. Furthermore, the technology is providing a low-cost, low-volume and low-risk route to market for entrepreneurs with novel products leading to a reduction in time to market for new innovations.

In order to meet the demands of entrepreneurs, designers and artists wishing to create ever more complex and high-tech products using 3DP technology, there is a move towards the incorporation of functional elements such as electronic sensors into 3D printed macroscale structures. To achieve this goal, low-cost, easy to use functional materials and 3D printing methodologies are required.

Here we present a new paradigm in 3D printing technology with the formulation of a new simple, low-cost conductive composite material (*termed ‘carbomorph’*) from easily available starting materials. The material is used in conjunction with a low-cost Bits from Bytes BFB3000 3D printer to produce a range of functional sensors as either standalone devices or embedded as part of a 3D printed structure. We demonstrate how the piezoresisitve nature of the conductive composite can be used to sense mechanical flexing when either added to an existing object or for example embedded into an ‘exo-glove’ interface device for sensing the flexing of a hand. Furthermore, we demonstrate how the material can be used to create capacitive sensing devices for custom 3D printed Human-Interface-Devices (HIDs) and to create embedded capacitive sensors to produce smart vessels which are able to sense the amount of liquid placed inside. The printed sensors are simple to interface to and require no complicated electronic circuits or amplification, in-fact the sensors can be monitored using existing open-source electronics and freely available programming libraries. Standard print settings were used and no modifications to the printer were required. A significant advantage in using 3D printing to create electronic components such as these is that sockets for connecting to standard equipment such as interface boards and multimeters can be printed as part of the printed structure whereas a 2D printed electronics approach using a technology such as inkjet printing would require the use of conductive glues and paints. This approach will open up many new applications for 3DP where fully interactive devices can be printed, for instance, designers could understand how people tactilely interact with their products by monitoring sensors embedded inside.

## Results and Discussion

### Material Formulation and Testing

In order to formulate a conductive material for use with the BFB3000 3D printer a conductive Carbon Black (CB) filler was chosen. CB is an amorphous form of carbon, produced from the incomplete combustion of heavy petroleum products such as FCC tar, coal tar, ethylene cracking tar and a small amount from vegetable oil. As such it is a readily available and inexpensive. Amorphous CB has been previously shown to be a good filler material in conductive polymer composites. [11]. CB is preferable for this application over a material such as copper because finely divided copper is prone to oxidation and becoming non-conductive. A transition from insulating to non-insulating behaviour for composites with a conductive filler is generally observed when the volume concentration of filler reaches a threshold of about 25%. [12]. To provide a printable thermoplastic matrix for the composite, we chose a readily available modeling plastic, polymorph, a commercial formulation of polycaprolactone (PCL). PCL is a biodegradable polyester with a low melting point of around 60°C and a glass transition temperature of about −60°C. The low temperature processing conditions of the polymorph offers significant advantages for formulating the final composite to work in the 3D printer as it did not require high temperature or expensive extrusion equipment. We termed this new composite material ‘carbomorph’.

In choosing a filler ratio we considered both the percolation threshold and the melt viscosity of the composite. The electrical conductivity of the of the carbomorph depends on the physical mixture of the conductor with the insulator in high enough proportions that electrons can either tunnel or percolate through a network of carbon black. The filler ratio had to be high enough to deliver a useable electrical conductivity but low enough so as to enable the material to exit the heated extrusion nozzle of the 3D printer. This creates a unique situation requiring carbon loading near the limits of the polymer processing conditions. The appropriate loading of CB was chosen through tests to observe how the composites performed under extrusion through the printer nozzle.

**Figure 1:**
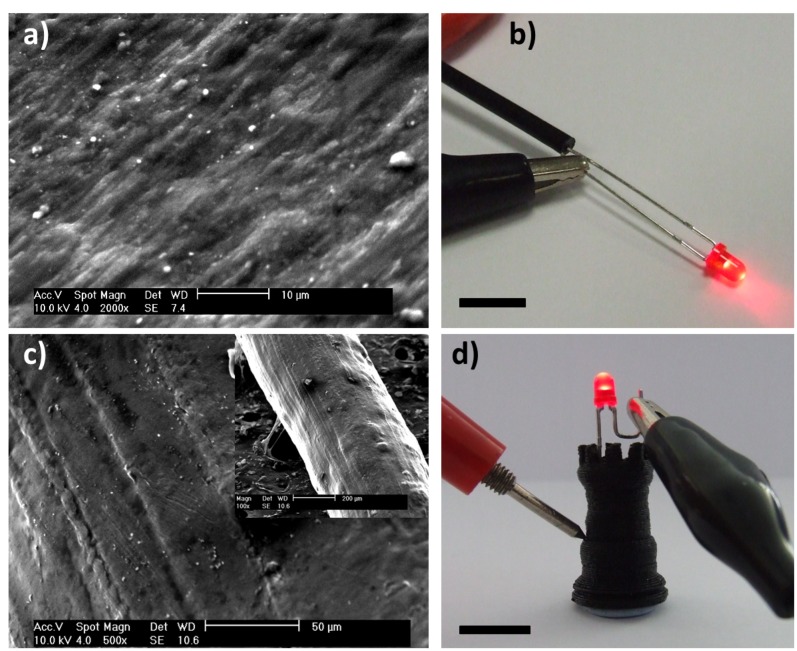
Characterisation of the conductive composite material produced. *a)* an SEM image of a cut edge of the formulated conductive material, *b)* photograph showing a length of the composite being used to connect to an LED, (scale bar 5 mm) *c)* Large scale SEM image of the conductive material after passing through the 3D printer nozzle (inset) a reduced magnification SEM image showing the extruded material, *d)* photograph of 3D printed chess rook also being used to light an LED (scale bar 10 mm).

The final chosen loading of CB in the composite was 15wt%. This value falls above the literature value for the percolation threshold in carbon black polymer composites [13]. Higher loadings of CB gave a composite that was unable to pass through the standard heated nozzle of the 3D printer and required the nozzle to be drilled out to 1.5 mm diameter and prints to be carried out at 260°C and above, significantly compromising print resolution. Density measurements of the unmodified polymorph polymer and the carbon black were carried out using a helium pycnometer and revealed the densities to be 1.1505 and 2.47 g/cm^3^ respectively. Scanning Electron Microscopy (SEM) of the carbomorph filament was carried out with no pre-coating to enhance conductivity. SEM images show the presence of some CB particles visible on the cut material surface. Beyond this, no large-scale aggregates were observed, suggesting a well-dispersed nanoscale CB filler (Fig. 1a).

The formulated composite filament is pictured in figure 1b. To demonstrate the presence of electrical percolation in the composite, a filament of carbomorph 3 mm feedstock material was incorporated into a simple electronic circuit and used to pass current to an LED. An SEM image of the extruded material can be seen in figure 1c. The presence of carbon particles on the surface of the composite can be seen along with striations along the length of the material. These striations are believed to arise from microscale roughness inside the print nozzle. The carbomorph was used for printing a 3D chess rook test structure which was also used to light an LED (Fig. 1d). The printed composite was tested for its resistivity both in-plane of the printed layers and perpendicular to the layers. Tests for resistance were carried out on 5 mm cubes of carbomorph using a two-probe measurement with the two opposite cube faces painted with silver conductive paint to minimise contact resistance. The measured resistivity of the composite in-plane with the layers was 0.09±0.01 ohm m^−1^. Perpendicular to the layers, the resistivity was 0.12±0.01 ohm m^−1^. A reduction on the resistivity of 25% was encountered when moving from the perpendicular resistance to parallel resistance mode. The difference in resistivity is explained by the way in which the blocks were printed. In the plane of the layers, the printed filaments provide a complete conductive path between electrodes. Perpendicular to the layers the establishment of a conductive pathway is reliant upon melting between printed layers. Current-voltage (IV) analysis was carried out on the printed composite cubes in both orientations between −5 and +5 V using a potentiostat and showed the IV response in both orientations to be linear.

### 3DP Flex Sensor

**Figure 2:**
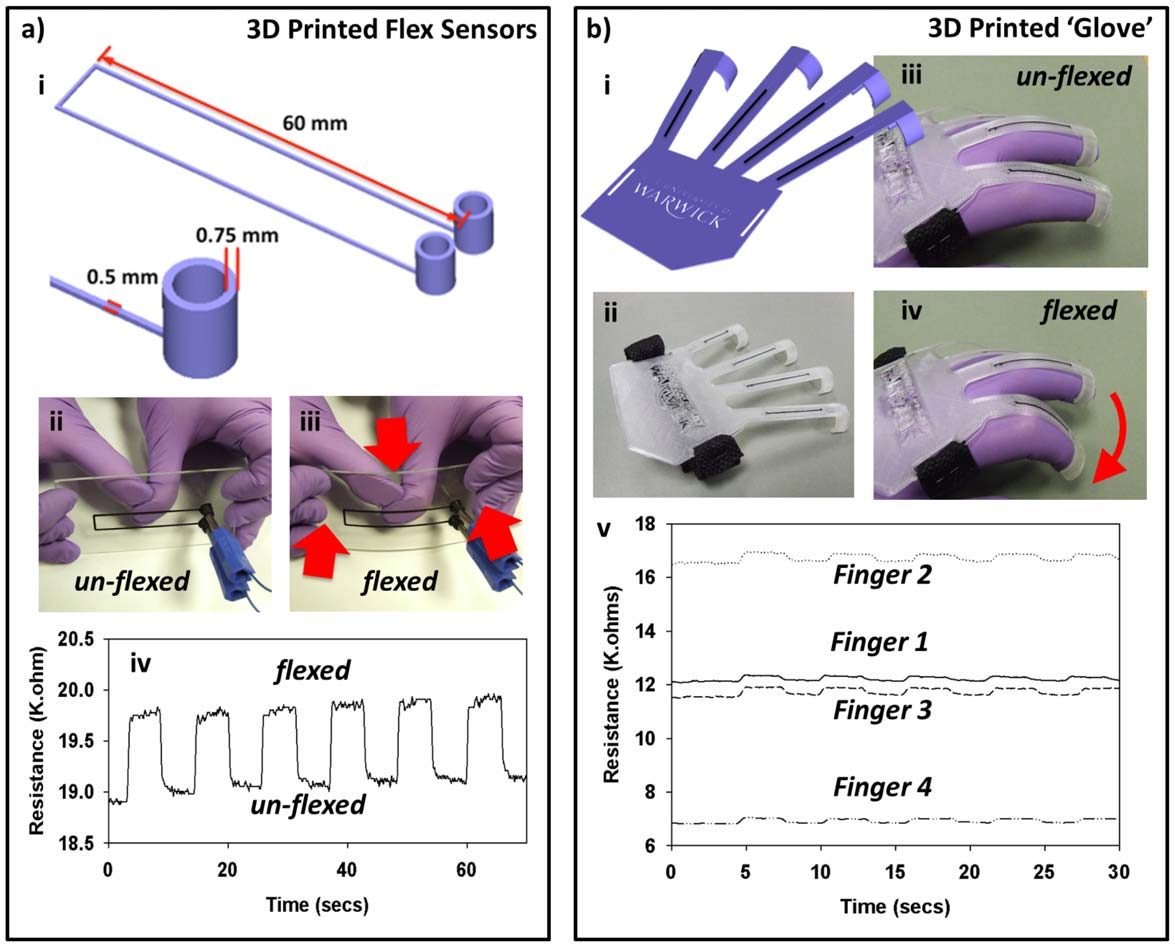
3D printing of flex sensors. *ai)* the CAD design of flex sensor, *aii)* the printed flex sensor, *aiii)* the printed sensor undergoing flexing, *aiv)* the resistance response of the sensor during flexing, *bi)* CAD design of the 3D printed ‘glove’, *bii)* the printed ‘glove’, *biii)* the printed ‘glove’ before flexing, *biv)* the printed ‘glove’ during flexing and *bv)* the resistance response of each finger during 5 flexings.

During resistivity tests, it was noted that the printed carbomorph material exhibited piezoresistive behaviour. The piezoresistive effect describes the changing resistivity of a semiconducting material due to applied mechanical stress. Piezoresistivity is a common sensing principle for micromachined sensors [14]. Doped silicon for example exhibits a piezoresistive response to mechanical manipulation. Piezoresistive materials have been used for the production of pressure sensors or mechanical stress sensors, however, the fabrication of such devices still requires multiple processes or the use of conventional silicon technology [15]. In order to test the sensing properties of the printed material and incorporate the electrical connection method into the devices, a CAD design was made of a standalone monolithic printed device composed of a carbomorph track (composed of a single printed filament) with two printed sockets at the end for connection to ‘banana plugs’ (Fig. 2ai).

By printing the sockets for connection in the same single build process the need for connecting to the tracks using conductive paint or glue for instance is removed, making sensor implementation and use much easier. The sensor was printed onto a sheet of 2 mm perspex and the cross-section of the sensing track measured using a surface profilometer. The thickness of the printed track was 240 µm at its highest point, with the planned thickness being 250 µm. The width of the track was measured at 1 mm which was wider than designed, however this was believed to be due to spreading resultant from the lower melt viscosity of the carbomorph compared to the standard printing materials. The printed sensor was connected using ‘banana plugs’ and single core copper wire to an arduino electronics prototyping platform for data capture through a potential divider to measure resistance (Fig. 2aii). Upon minimal flexing of the perspex sheet by hand (Fig. 2aiii) a change in the resistance of the sensor was measured (Fig. 2aiv). The change observed was a 4±0.13% change and could be repeated over 50 times. The ability to 3D print sensors in this fashion has fascinating implications for sensors to be rapidly produced in a bespoke fashion *in-situ* on structures for structural health monitoring.

### 3DP Embedded Flex Sensor

Piezoresistive strips produced using carbon nanotubes have previously successfully been employed in the measurement of human movement [16]. In order to examine whether 3D printed sensors could be used to carry out the same task, an ‘exo-glove’ was designed consisting of a main body of 3D printed clear PLA and strips of carbomorph embedded over each finger to detect resistance changes upon movement of the finger (Fig. 2bi). The design incorporated loops for attachment of a securing a strap and printed jaws for gripping to fingers. The whole device was printed in a single, un-paused print run with the flat back portion of the ‘glove’ printed first (Fig. 2bii). Contacts were made to the tracks in this case using silver loaded epoxy resin in order to maintain an ohmic contact during testing. Future versions of the glove could include the printed sockets for connection as seen in section 2.2. Upon the authors putting on the glove and flexing a finger (Fig. 2b iii & iv) the resistance of the tracks was seen to change. This effect could be repeated for each of the fingers of the glove (Fig. 2bv). A difference in initial resistance of the individual fingers was observed due to the differing length of the carbomorph tracks within each finger. Fingers 1 and 3 (the index and ring fingers respectively) are of similar length, while finger 2 (the middle finger) is much longer and hence exhibits a higher resistance and finger 4 (the little finger) is shorter and hence exhibits a lower resistance. Each finger was flexed 5 times with the resistance seen to increase upon flexing. The magnitude of the response is much less than conventional piezoresistive materials used in flex sensors, however, the amount of sensing material present is quite small (cross-section of approximately 0.25 µm^2^) and could be increased if required to boost sensor response. With the manufactured sensors, the response was still detectable with a simple potential divider and basic electronics and did not require amplification. The strength of the 3D printing technique is being able to use smaller amounts of material only where they are required, hence reducing material waste. Such printed devices could be used in the field of biomechanics for printing of bespoke patient-tailored sensors to aid in their rehabilitation after accidents.

### 3DP Capacitive Buttons

**Figure 3:**
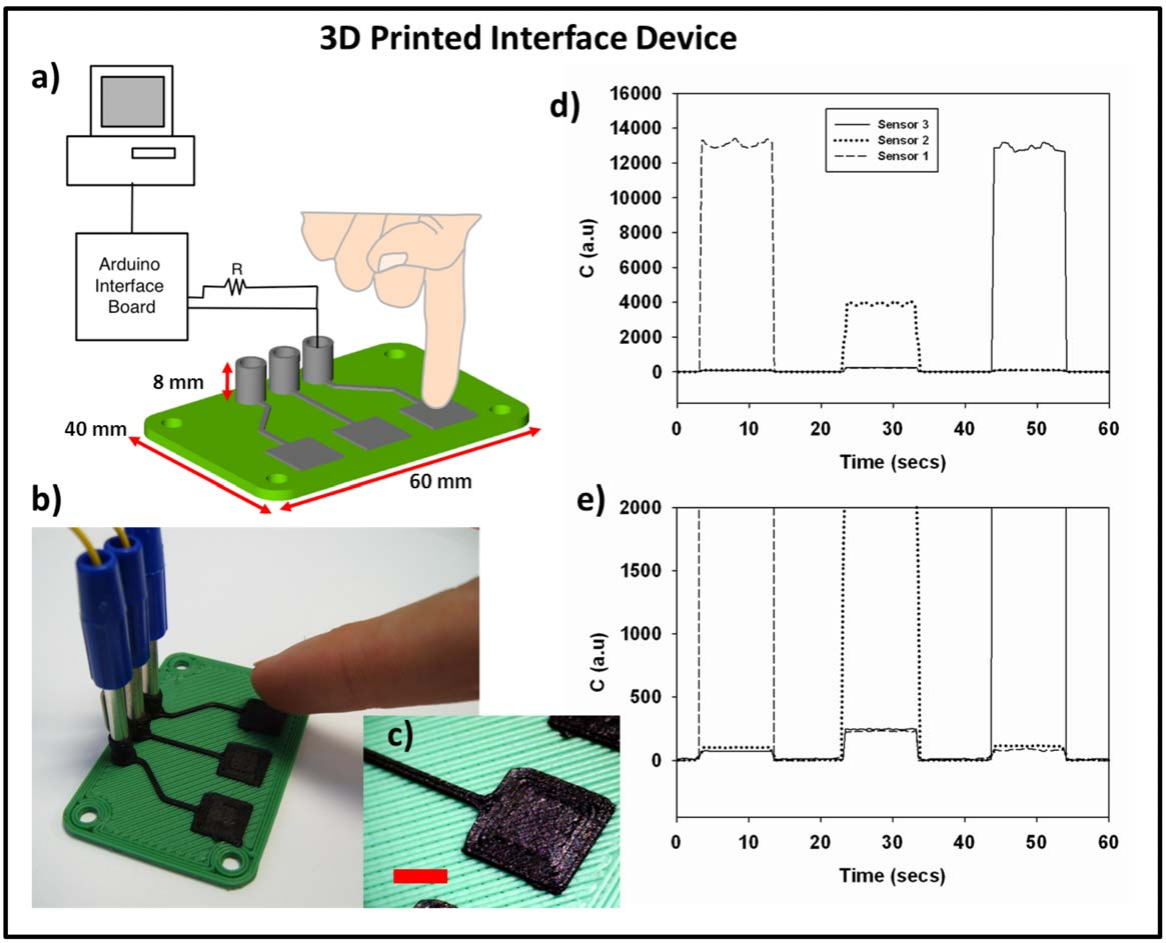
3D printing of capacitive interface device. *a)* the CAD design of the printed interface device and the simple circuit used to detect inputs, *b)* a photograph of the printed device, *c)* a macro image of the printed sensor pads (scale bar 5 mm), *d)* the capacitance of each printed sensor pad plotted against time *e)* an enlarged portion of the graph from part d showing the cross-sensitivity of each sensor pad.

In addition to it's piezoresistive properties, the carbomorph could be used to print capacitive devices. Capacitive sensing is used in many different types of sensors, including those to detect and measure proximity [17], position [18] or displacement [19], humidity [20], fluid level [21], and acceleration [22]. Capacitive sensing as a Human Interface Device (HID) technology, for example to replace the computer mouse, is growing increasingly popular. An example 3D printed capacitive interface was designed to interface to a computer as described in figure 3a. Again, the device was designed to accept commonly available ‘banana plugs’ into a 3D printed socket. To use the printed device, a user touches the printed conductive pad, the capacitance of the pad increases, which is then sensed by the arduino board and used to trigger an operation.

The capacitive HID was implemented using the arduino CapSense code library. The CapSense arduino library uses a pair of IO pins on the arduino interface board as a capacitive sensor. The circuit requires a high value resistor and a connection to a sensing pad, in this case the 3D printed device. When an IO pin on the arduino changes state (termed the send pin), it will effect a change in state of a second connected IO pin (termed the receive pin). The temporal delay between the change in state of the send pin and the change in state of the receive pin is determined by an RC time constant, defined by R×C, where R is the value of a resistor at the send pin and C is the capacitance at the receive pin, including any other capacitance present at the printed sensor pad. The complete 3D printed capacitive HID is presented in figure 3b and shows the complete device with connected circuit plugs. Figure 3c shows a macro image of the printed sensor pad demonstrating the print quality achievable. Often when using filled composite materials for 3D printing procedures a compromise is required with printed resolution due to working close to the limits of material processability. No such negative impact on resolution was seen here when compared to the supplied standard printing materials.

The ΔC values for each pad when being pressed using a fixed circuit resistor of 10 MΩ are presented in figure 3d. Sensor 1 was pressed first, followed by number 2 and 3. It can be seen that the sensor response to being pressed is quite considerable. Figure 3e shows an enlarged region of the same graph showing that a small amount of cross-sensitivity is seen between sensors, however when compared to the capacitance change upon being pressed, this cross-sensitivity is quite small (2% of ΔC for button 2). This cross-sensitivity could be overcome by use of extra capacitors in the circuit and printing of a ground plane within the device.

### 3DP ‘Smart’ Vessel

**Figure 4:**
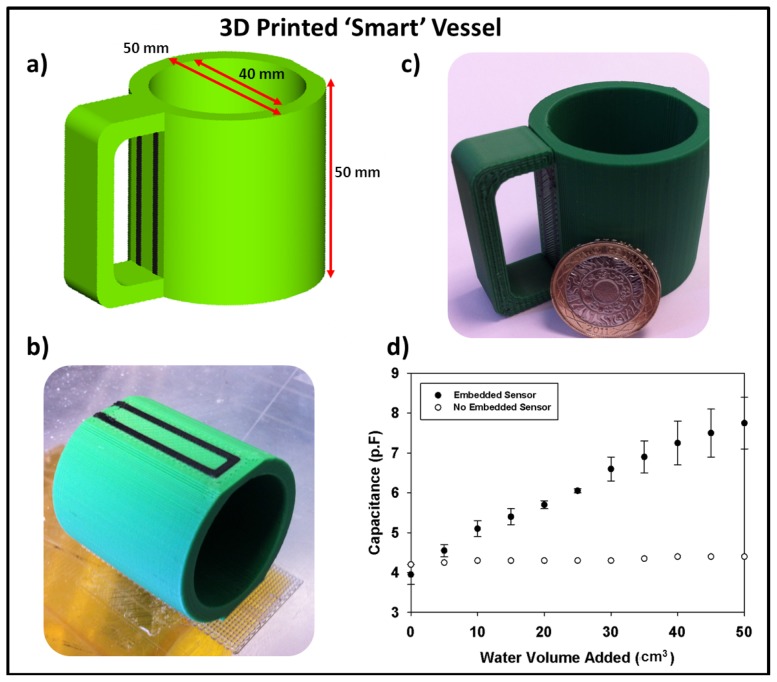
3D printing of capacitive ‘smart’ vessel. *a)* the CAD design of the printed ‘smart’ vessel, *b)* the vessel during printing showing the embedded sensor strip, *c)* the completed vessel next to a £2 coin (coin is approximately 28 mm in diameter) and *d)* the capacitance response of the ‘vessel’ when water is added.

With the ability to produce capacitive sensors along with 3D printed structures, other applications can be found for the technology. In order to demonstrate how this could be envisaged, a 3D printed mug was designed and created containing two conductive tracks in the sides of the mug to create a ‘smart vessel’ (figure 4a and b). For testing, two self-adhesive copper tape pads were placed on the base of the cup and connected *via* soldered wires to a capacitance meter. On filling of the cup with water there was minimal change in capacitance. The copper tape pads were then connected to the embedded sensor using silver paint and the process repeated. Upon filling the mug with water for the second time the capacitance was seen to vary in an approximately linear relationship to the added volume of water. This process demonstrated that sensors placed through the entire height of the vessel (and in this case embedded in the side walls of the vessel) were able to detect the presence of the liquid as opposed to just the copper pads.

The ability to embed sensors such as these in printed objects could allow artists and especially designers to understand how people interact with printed sculptures or objects. The technology could also help in the production of medical devices incorporating biosensors or the implementation of sensors into objects to make them smarter and more functional.

## Conclusions

The formulated material has enabled the rapid production of a range of functional electronic sensors using a simple, low-cost 3D printer. The sensors range from piezoresistive sensors able to sense mechanical flexing when either placed on an existing object or embedded inside a printed object, through to capacitive sensors printed as part of custom interface device or embedded inside a ‘smart’ vessel able to sense the presence and quantity of liquid inside.

Overall we have demonstrated that rather than just being a technology for producing benign, non-functional structures, 3DP when combined with capable, functional materials is able to produce far-more functional objects incorporating electronic sensors that can be used in a number of ways. This advance has exciting possibilities for a number of fields and applications ranging from medical implants through to creative industries. This material offers individuals the ability to produce complex products incorporating high-tech sensors on a low-cost, desk-top printer without the need for complex circuit and sensor production facilities.

## Materials and Methods

The 3D printer used was a triple-head BFB3000 purchased from Bits from Bytes Ltd (Clevedon, UK). Green ABS (Acrylonitrile butadiene styrene) and clear PLA (Poly(lactic acid)) printing filament were also purchased from Bits from Bytes Ltd and used as received. Conductive filament (termed ‘carbomorph’) was formulated using a conductive carbon black filler (Cabot Corp, Black Pearls 2000) in a matrix of a commercial formulation of polycaprolactone (Polymorph, Rapid Electronics, UK). CAD designs were drawn and visualised in TurboCAD for Mac and transferred to 3D printable format using the Axon 2 software supplied with the BFB3000.

### Conductive Filament Formulation

To produce the carbomorph filament, 3 g of the polymorph thermoplastic was added to a stirred suspension of carbon black in 40 ml of dichloromethane. Stirring was continued for 1 hour. After stirring the suspension was poured onto a glass watch glass and the DCM allowed to evaporate in a fume hood for 1 hour. The resultant composite film was placed in a water bath at 80°C for 1 minute then removed and rolled between two glass plates. The warming and rolling was continued until a 3 mm wide filament of carbomorph was achieved. The filament was then left to cool for 2 hours before further use. 3D printing was carried out using standard print settings with no modifications to the printer. The carbomorph was printed using the print settings for standard PLA. Density measurements of the polymorph polymer and the carbon black were carried out using a helium pycnometer (AccuPyc II 1340, Micromeritics, UK). Current-voltage (IV) analysis was carried out on the printed composite cubes in both orientations between −5 and +5 V using an Autolab PGSTAT30 potentiostat (Metrohm Autolab, NL). SEM imaging was carried out without any prior treatment to enhance conductivity.

### Resistive Measurements

Resistivity measurments were carried out on 5 mm cubes of carbomorph using a two-probe measurement (Solartron 7075 Digital Voltmeter) with the two opposite cube faces painted with silver conductive paint (Electrolube, RS Components, UK) to minimise contact resistance. Piezoresistive measurements were carried out using an arduino Uno interface board purchased from oomlout.co.uk and captured using a program written in the Processing programming language (http://www.processing.org)

### Capacitive Measurements

Capacitive measurements were carried using either an arduino Uno implemented using the CapSense library (http://www.arduino.cc/playground/Main/CapSense) by Paul Badger or a Megger B131 LCR Meter (RS Components, UK).

### Supporting Information

The CAD files and 3D printer build files used in this study are available for download from go.warwick.ac.uk/msl/CADdata
